# Leveraging Supervisor Knowledge Sharing Behavior and Organizational Absorptive Capacity on Nurses' Creativity

**DOI:** 10.1155/2024/5480761

**Published:** 2024-03-12

**Authors:** Ahmed Abdelwahab Ibrahim El-Sayed, Nariman Ahmed Mohamed Elbassal, Samira Ahmed Alsenany, Sally Mohammed Farghaly Abdelaliem

**Affiliations:** ^1^Nursing Administration Department, Faculty of Nursing Alexandria University, Alexandria, Egypt; ^2^Department of Community Health Nursing, College of Nursing, Princess Nourah bint Abdulrahman University, P.O. Box 84428, Riyadh 11671, Saudi Arabia; ^3^Department of Nursing Management and Education, College of Nursing, Princess Nourah bint Abdulrahman University, P.O. Box 84428, Riyadh 11671, Saudi Arabia

## Abstract

**Background:**

Nurses' creativity is an imperative necessity for healthcare organizations to grow. The creative abilities of nurses are affected by many factors; some of these factors are stimulants and some of these are inhibitors. Supervisors' knowledge sharing behavior and the capacity to absorb new knowledge and technologies are important factors in the recent era that delineate the creativity of nurses. *Aim of the Study*. Assess the effect of supervisor knowledge sharing behavior and organizational absorptive capacity on nurses' creativity.

**Method:**

Cross-sectional multicenter descriptive correlational exploratory research design was used to conduct the study. Data were collected conveniently from 700 nurses recruited from five large hospitals at Alexandria, Egypt, using three self-administered printed questionnaires. Findings were investigated via descriptive and inferential statistics as well as structured equation modeling.

**Results:**

Nurses' creativity was positively associated with supervisor knowledge sharing behavior and organizational absorptive behavior (*r* = 0.619, *p* value <0.001, and *r* = 0.545, *p* value <0.001, respectively). Supervisor knowledge sharing behavior accounted for 87% of variance in nurses' creativity, while organizational absorptive capacity accounted for 55% of variance in nurses' creativity.

**Conclusion:**

Supervisors' knowledge sharing behavior and absorptive capacity of organization are powerful significant stimulants for nurses' creativity. *Implications to Nursing Management*. Hospital directors, managers, and nursing leaders should cultivate knowledge sharing behavior in healthcare settings by establishing a reward and incentive system for healthcare workers who share their knowledge with their colleagues for mutual benefit and organizational development.

## 1. Introduction

The economic decline and inflation around the world have been affecting healthcare in all its aspects, which prompted organizations to attract cadres from creative health care providers[1]. Moreover, the sustainability and competitiveness of healthcare organizations in the contemporary highly turbulent economic environment articulated around three critical attributes, namely, organizational culture that motivate leaders to share their experiences and knowledge in a successful manner, absorptive capacity of organization, and the ability of organization to recruit creative and innovative nurses who are able to use different sources of knowledge to provide efficient integrated nursing care [2].

Today, financial and technological resources are not the only advantages of healthcare organizations. It is important to have creative nurses who can compensate for the lack of other resources [3]. Understanding the strategic role of human resources especially nurses in making excellence is one of the value added features of successful healthcare organization. Only successful organizations are able to delineate stimulants and barriers of creative performance of their nurses. Numerous antecedents and factors can stimulate nurses creativity; however, knowledge sharing behavior exhibited by their leaders as well as the absorptive capacity of organizations to invest in novel knowledge sources and technology modalities are two factors responsible for the majority of creative ideation among nurses [4].

Knowledge sharing is a corner stone process as no one person cannot generate all the knowledge needed to carry out his/her everyday tasks [5]. Everyone must therefore acquire outside knowledge and experience [6]. According to Che et al. [5], individuals within organizations, including supervisors, can impart their knowledge and experience on others. Supervisors can also help their subordinates improve their work by imparting knowledge or useful information [7]. The value of knowledge and experience in creative performance has been demonstrated in the prior studies [8, 9]. Supervisor knowledge sharing behavior is regarded as prosocial behavior that is beneficial to nurses. When supervisors share their valuable knowledge, experts, and skills with nurses, they are likely to feel appreciated and learn this behavior [10]. Firms should choose individuals who frequently share their knowledge with others when staffing supervisory positions. Moreover, healthcare firms should encourage nursing supervisors as well as give supervisors a substantial amount of time and opportunities to share their knowledge with nurses [4].

Explicit knowledge and tacit knowledge are the two categories of knowledge [11]. Objective knowledge that can be expressed, codified, and conveyed using formal language is known as explicit knowledge. Tactic knowledge is described as subjective knowledge that is challenging to articulate, codify, and transfer [11]. Although it is challenging to convey tacit knowledge, supervisors can do so through interacting with, socializing with, and training their subordinates [12]. In addition, networking with others and face-to-face conversation can lead to information sharing [13]. Sharing information and experience with subordinates can be beneficial for supervisors. The ability to think creatively depends on knowledge [8, 14]. Supervisors can work with others to create and implement new ideas or procedures as well as exchange task-relevant ideas, knowledge, experience, and suggestions with subordinates [12]. Che et al. [5] asserted that employees' creative performance would be enhanced by learning from sources within organizations such as supervisors and leaders.

Absorptive capacity is the dynamic capacity that allows organization to create value, gain, and sustain a competitive advantage through the management of the external knowledge [15]. It is the ability of the organization to identify technological opportunities in its external environment and apply them to obtain a better performance [16]. Healthcare organizations can upgrade their absorptive capacity by mastering knowledge management skills, especially knowledge acquisition, learning, and assimilation systems. If these organizations need to shape their creativity strategies, they not only need to absorb new information from the environment but also use it internally [15].

Knowledge sharing behavior of supervisors and absorptive capacity are two concepts that are closely related. The relationship between them is interdependent and complementary. Knowledge sharing of supervisors can be seen as an antecedent of absorptive capacity because it provides the necessary knowledge for absorptive capacity to occur. On the other hand, absorptive capacity can be seen as an antecedent of knowledge sharing because it provides the necessary context for knowledge sharing to occur [17].

Koseoglu et al. [18] described creativity as the development of novel, useful products and processes. Creativity is essential for organizational innovation, performance, growth, and long-term survival. Every nurse has a creative mode, but it is frequently inhibited by internal and external variables including time, overly rigid control systems, and an excessive dependence on standard operating procedures [19]. Creativity is imperative for nursing practice as when there are no clear answers or courses of action, nurses can rely on their creativity to come up with new solutions and make decisions. In this context, the work of Zuber and Moody [20] highlighted that creativity in nursing care includes fluidity of mind and the creation and acceptance of new ideas for patient care in such a way that the new methods are simple, useful, efficient, affordable, and safe.

When it comes to creativity, knowledge sharing can benefit creativity through absorptive capacity and knowledge integration. Thuan [21] found that knowledge sharing was positively related to team creativity, fully mediated by both absorptive capacity and knowledge integration. In addition, cognitive team diversity played a moderating role in the relationship between knowledge sharing and organizational absorptive capacity, as well as in the relationship between knowledge sharing and knowledge integration [3].

### 1.1. Significance of the Study and Research Gap

Stimulating nurses' creativity is of paramount importance for healthcare organizations and nursing practice [1]. Recent studies stressed on the promising role of nurses' creativity toward buffering resources depletion and giving organization high magnetism power that enable it to attract patients and qualified cadres [2, 22]. This also strengthens organization in the pursuit of competitive advantage and sustainability [23]. Moreover, studying factors that stimulate creativity is highlighted in different scholars as recent required research direction due to its powerful role in transforming the conventional nursing practice and making it smart, flexible, green, evidence-based, patient-centered, and holistic practice [1]. Meanwhile, international council of nurses dedicates its efforts to shape nursing policy in the sustainable developmental era highlighting the importance of investment in nursing creativity [2]. Despite all these calls, nurses' creativity does not receive considerable attention in recent studies. The majority of studies that addressed creativity articulated around perspectives of nurses regarding their creativity. In addition, stimulants and barriers of nurses' creativity are not adequately explained with limited studies that shed the light on the role of different factors like clinical practice environment [3], career development, job embeddedness [24], and polychronicity [25] on nurses' creativity in the Egyptian and international contexts.

The concepts of supervisor knowledge sharing behavior and absorptive capacity have gained high momentum recently in knowledge management literature [15]. Several studies examined their role in shaping innovation and employees' performance at different contexts like tourism [26], information technology agencies [21], public services settings [27], and petroleum companies [8]. Meanwhile, opinions of respected nursing authorities give consensus that supervisor knowledge sharing behavior and organizational absorptive capacity are important factors to build creativity among nurses [28]. However, these factors were not tested empirically in nursing context. To address this gap, our study aims to empirically examine the role of supervisor knowledge behavior and absorptive capacity toward nurses' creativity. Investigating the interplay among these factors could help nurse leaders to develop trusted strategies for cultivating creative work environment for nurses which ultimately improves quality of patient care and eradicate patient safety threats.

### 1.2. Theoretical Framework and Hypothesis Formulation

**Figure 1:**
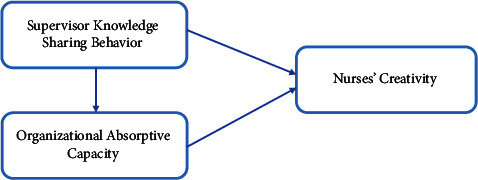
Conceptual model illustrating the relationship between study variables.

This study combines organizational learning theory and organization knowledge management model developed by Vespo [29] to explain the relationship between knowledge sharing behavior, absorptive capacity, and nurses' creativity. According to organizational learning theory, organizational learning occurs through acquiring new knowledge shared from past experiences and absorbing new knowledge from outside organizations which could reinforce using absorptive capacity strategies [30]. Organizational learning theory believed that organizational learning is the behavioral process of organization employees to promote innovation and creative ideation using knowledge sources gained by sharing and absorbing [15]. In addition, the theory also assumed that fertilizing the land for creativity and innovation to grow require the organization to mix between both adaptive learning based on environmental change response and active learning based on self-motivation. In this respect, Vespo [29] uses the assumptions of organizational learning theory and develops simplified model illustrating organizational knowledge management. Accordingly, knowledge management starts with identifying new knowledge sources through knowledge sharing which in turn lead to elaboration through combining different skills with each other. The Result of this elaboration is in the first place the outcome itself, which may be creation or an innovation or an improvement of an existing service [29]. Our study uses the assumptions of organizational learning theory and the work of Vespo to conceptualize and predict the relationship between the three variables.  Figure 1 shows our proposed conceptual framework illustrating the relationship between knowledge sharing behavior of supervisor, organizational absorptive capacity (independent variables), and nurses' creativity (dependent variable).

### 1.3. Study Hypotheses

 
*H1*. There is a relationship between supervisor knowledge sharing behavior and nurses' creativity
 
*H2*. There is a relationship between organizational absorptive capacity and nurses' creativity
 
*H3*. Supervisor knowledge sharing behavior has positive effect on stimulating nurses' creativity
 
*H4*. The level of organizational absorptive capacity has positive effect on stimulating nurses' creativity

## 2. Methods

### 2.1. Aim

The study aims to investigate the effect of supervisor knowledge sharing behavior and organizational absorptive capacity on nurses' creativity.

### 2.2. Research Question

- To what extent nursing supervisors share their knowledge with nurses?
- What is the level of organizational absorptive capacity perceived by nurses?
- What is the level of creativity among nurses?
- Is there a relationship between supervisor knowledge sharing behavior and nurses' creativity?
- Is there a relationship between the level of absorptive capacity of organization and nurses' creativity?

### 2.3. Design

A cross-sectional multicenter, correlational, and descriptive research study design was used.

### 2.4. Study Settings

This study was carried out at five hospitals in Alexandria, Egypt: the Alexandria Main University Hospital, the Gamal Abd-Nasser Hospital, the Sharq El Medina Hospital, the Al Gomhoreya Public Hospital, and the Mabaret Alasafra Hospital. These hospitals are affiliated to the University, the Health Insurance Organization, the General Secretary of Specialized Medical Centers, Ministry of Health and Population, and Private sectors, respectively. Each of the selected hospitals was selected as it is considered the largest capacitated one in terms of nurses' number and bed capacity. Moreover, these hospitals participate actively in “innovate Egypt” program that was launched by the Egyptian government to ultimately connect educational services for enhancing healthcare-related knowledge with community requirements in different healthcare directions through empowering the next generation of Egyptian innovators that include different healthcare providers (e.g., nurses, physicians, technicians, and so on).

### 2.5. Study Participants and Sampling

A straightforward sampling by power analysis technique was used to pick a sample of 700 nurses (*n* = 700) from the total population of 1510 nurses in the chosen settings: 1510 total subjects, 95% confidence level, 5% margin of error, problem prevalence is 50%, and a maximum sample size of 700. It was determined that 20 individuals would be sufficient to conduct a reliable regression analysis because 20 participants were required for each study variable.

### 2.6. Study Measurements

Demographic data including age, gender, qualification, unit, current position, and years of experience were collected using personal and work-related data questionnaire.

### 2.7. Supervisor Knowledge Sharing Behavior

To assess supervisor knowledge sharing behavior, we developed a tool based on literature review. It consists of 11 items: five items adapted from Thuan [21] and six items adapted from Nifadkar et al. [31]. The items grouped into two dimensions, namely, supervisor explicit knowledge sharing behavior (8 items, Cronbach alpha, *α* = 0.766) and supervisor tacit knowledge sharing behavior (3 items, *α* = 0.809). Participants rated each item on a five-point scale (1 = strongly disagree to 5 = strongly agree). We calculated the average score for each dimension, and the total scale score (Cronbach alpha, *α* = 0.861) was the average of the two dimensions; higher scores indicate that nursing supervisors have a high level of knowledge sharing behavior. Supervisors' levels of knowledge sharing were classified as follows: less than 50% for low level, 50% to less than 75% for moderate level, and 75% for high level.

### 2.8. Organizational Absorptive Capacity

We used the organizational absorptive capacity questionnaire for assessing absorptive capacity from the perspective of nurses [32]. This scale consists of ten items. We computed the average score for the total scale score using Cronbach alpha, *α* = 0.900. Responses were measured on a five-point Likert scale ranging from 1 = strongly disagree to 5 = strongly agree. The overall score level ranges from 10 to 50. Higher scores indicate high level of absorptive capacity. The levels of absorptive capacity of organization were arranged as follows: less than 50% for low level, from 50% to less than 75% for moderate level, and ≥75% for high level of absorptive capacity.

### 2.9. Nurses' Creativity

To assess nurses' creativity, we used the creativity self-assessment questionnaire [33]. It consists of 28 items. The items grouped into 4 dimensions, namely, generating ideas (7 items, *α* = 779), digging deeper into ideas (7 items, *α* = 736), exploring ideas (7 items, *α* = 792), and listening to the inner voice (7 items, *α* = 710). This tool demonstrated acceptable reliability where Cronbach alpha, *α* = 0.73, 0.771 in the studies of Adyasha and Duraipandian [34] and Naqvi et al. [35], respectively. We computed the average score for the total scale score (Cronbach alpha, *α* = 0.939). Responses were measured on a five-point Likert scale ranging from 1 = strongly disagree to 5 = strongly agree. The overall score level ranges from 28 to 140. Higher scores indicate a high level of creativity. The levels of nurses' creativity were defined as follows: less than 50% for low creativity, from 50% to less than 75% for moderate creativity, and ≥75% for high levels of creativity.

### 2.10. Study Tools Adaptation, Validity, and Reliability

#### 2.10.1. Tools Translation

Tools were translated from English versions into Arabic versions (see Supplementary 1) using the back-to-back translation technique [36] to adapt to Egyptian culture, ensure accuracy, and eliminate any potential threats to the study's validity.

**Figure 2:**
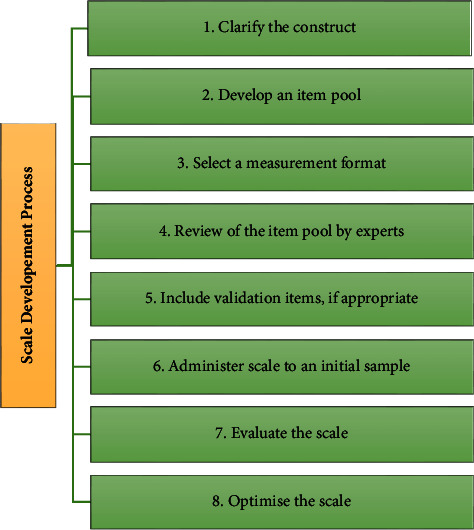
Steps in scale development [37].

Following the translation of the tools, we employed various methods to evaluate their validity and reliability, such as following Steps 1–6 of DeVellis' [37] model for scale development (see Figure 2) in relation to the coding instrument created. Clarification of the construct and development of an item pool were informed by a review of the literature review [21, 31]. In addition, content validity, exploratory factor analysis (EFA), confirmatory factor analysis (CFA), corrected item-total correlations, and Cronbach's alpha were applied. We used IBM SPSS software package version 22.0 and AMOS version 23.0 for the analyses (see Supplementary 2).

#### 2.10.2. Content Validity

A group of seven academics from the discipline includes three professors of information and data management and four professors of nursing administration. The panel identified word choice problems, typing errors, and punctuation errors. Several words were changed in response to their suggestions. In order to verify the accuracy and functionality of the instruments and determine the amount of time needed to complete study questionnaires, a pilot study with 70 nurses was also carried out and no modifications were made.

#### 2.10.3. Construct Validity

Both exploratory factor analysis (EFA) and confirmatory factor analysis (CFA) were used to assess the construct validity of the translated tools.

Kaiser normalization was used in conjunction with Promax rotation to conduct an exploratory factor analysis (EFA). The objective of the EFA was to pinpoint the fundamental elements or dimensions that account for the variation in each questionnaire item's response.

The EFA of the supervisor knowledge sharing behavior questionnaire revealed a clear and consistent factor structure that reflected the two dimensions of the scale with high boldface loadings for all items, ranging from 0.543 to 0.909 which means that the items strongly contribute to their factor. The Kaiser–Meyer–Olkin (KMO) measure of sampling adequacy was 0.703, which indicates the appropriateness of the data for factor analysis and that there is a high degree of common variance among the items.

The EFA of the absorptive capacity scale using Kaiser normalization revealed that there are two dominant factors that account for most of the variance in the responses. These factors have high loadings for all items, ranging from 0.617 to 0.747. This implies that it represents a general dimension of polychronic-monochronic tendency and that all items measure the same construct. The KMO value of 0.842 indicates that the data are very suitable for factor analysis, and that there is a high degree of common variance among the items.

The EFA of the nurses' creativity scale revealed a clear and consistent factor structure that reflected the four sections of the tool, with high loadings for all items on the corresponding factor, ranging from 0.522 to 0.756. This means that the items are more clearly nurses' creativity embeddedness. The KMO value of 0.853 indicates that the data are very suitable for factor analysis and that there is a high degree of common variance among the items.

#### 2.10.4. Confirmatory Factor Analysis

CFA was done using structural equation modeling (SEM) for the mean and standard deviation of the three scales used in the study (supervisor knowledge sharing behavior, absorptive capacity, and nurses' creativity). The CFA aimed to test the fit of the factor structure derived from the EFA to the data. The CFA confirmed that the model fits the data very well (comparative fit index (CFI) = 1.000, incremental fit index (IFI) = 1.000, root mean square error of approximation (RMSEA) = 0.117, Model *X*^2^ = 5.120, and *p*=0.001^*∗*^).

### 2.11. Reliability

The reliability of the tools was assessed using the corrected item-total correlations and internal consistency.

#### 2.11.1. The Corrected Item-Total Correlations

The corrected item-total correlations of the supervisor knowledge sharing behavior questionnaire showed that all items were positively and significantly related to their respective sections and to the overall score of the survey, ranging from 0.576 to 0.905. The corrected item-total correlations of the absorptive capacity scale showed that all items were positively and significantly related to the scale, ranging from 0.628 to 0.763. The corrected item-total correlations of the nurses' creativity scale showed that all items were positively and significantly related to their respective sections and to the overall score of the tool, ranging from 0.536 to 0.827. This means that all items are positively related to their respective subscales and that they measure the same construct as the other items in the subscale.

#### 2.11.2. Internal Consistency

Cronbach's alpha was used to measure the internal consistency of the tools. It is a measure of how well a set of items measures a single construct or dimension. It ranges from 0 to 1, with higher values indicating higher reliability and internal consistency. Cronbach's alpha values for the subscales of the supervisor knowledge sharing behavior ranged from 0.766 to 0.809 and 0.861 for the overall survey. Cronbach's alpha of absorptive capacity was 0.900. Cronbach's alpha values for all subscales of nurses' creativity scale ranged from 0.710 to 0.792, and the overall scale was 0.939. These results indicate acceptable reliability and internal consistency of the three study tools.

### 2.12. Data Collection

The administration of the hospitals officially approved the collection of data. By delivering the anonymous questionnaire (hand delivered), data were gathered over a three-month period, from the first of May 2022 to the end of July 2022. The questionnaire took around 25 minutes to complete and asked questions about participant demographics, supervisor knowledge sharing behavior, absorptive behavior, and creativity.

### 2.13. Data Analysis

Frequencies and percentages were utilized to illustrate the socio-demographic data; mean and standard deviation (SD) were employed to display continuous variables. The association between supervisor information sharing behavior, organizational absorptive capacity, and nursing creativity was examined using the Pearson correlation coefficient analysis (*r*). Inferential statistics (Pearson correlation coefficient and regression analysis (*R*^2^)) were used to examine the study's findings. A 0.05 alpha error was used for all statistical calculations. The mediating impact of career plateauing was investigated using a structural equation modeling approach. The moderator, organizational absorptive capacity, the independent variable, and the dependent variable, nurses' creativity, were examined by the researchers. All statistical analyses were performed using IBM SPSS Statistics and IBM SPSS AMOS versions 23 (Armonk, NY). The 0.05 level of statistical significance was used to determine the two-tailed nature of all given *p* values.

## 3. Results

Table 1 reveals that 47% of participants were within the age group 25–40 years with a mean score of 34.19 ± 10.16. Furthermore, near to two-thirds of the participants were female (61.6%) with a Bachelor of Science Degree in Nursing (48.4%) followed by Diploma Degree in Nursing (30.6%) with 49.6% of participant with years of experience less than ten years, followed by 26.9% of them with more than 20 years of experience. In relation to the current working unit, near to one-third of the participants were working in ICU, followed by 20.7 of the participants were working in ER in the following healthcare settings as the majority of them (45.7%) were working at Alexandria Main University Hospital, followed by Gamal Abdel Nasser Hospital (18.3), and both hospitals were public sector, while 15.35 of participants were working at Mabaret Alsafra Hospital, which is related to the private health sector.

Table 2 shows that most of the study participants had a moderate perception level regarding their supervisor knowledge sharing behavior, their organizational behavior regarding absorptive capacity, and creativity (67.4%, 74.7%, and 80.9%), respectively. The mean score (±standard deviation) of the supervisor knowledge sharing behavior (possible score range: 1–5) was 3.66 ± 0.43, and supervisor tacit knowledge sharing behavior had the highest mean domain score (3.91 ± 0.71). The mean score of absorptive capacity (possible score range: 1–5) was 3.62 ± 0.41. As well as, the mean score of creativity (possible score range: 1–5) was 3.64 ± 0.35, and generating ideas and digging deeper into idea domains had the highest mean scores (3.69 ± 0.48 and 3.69 ± 0.47, respectively).

Table 3 shows that nurses' creativity was positively associated with supervisor knowledge sharing behavior and absorptive behavior (*r* = 0.619, *p* value <0.001, and *r* = 0.545, *p* value <0.001, respectively).

**Figure 3:**
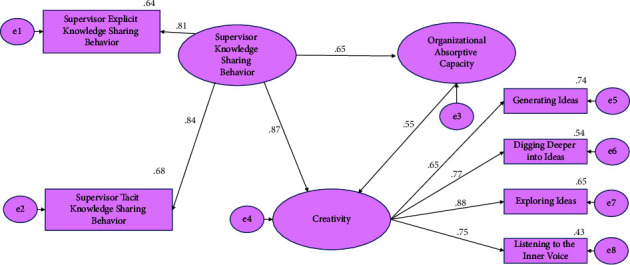
Path diagram with standardized parameter estimates.

Figure 3 depicts the structured equation modeling's standardized regression weights (Chi-square = 1145.530; degree of freedom = 15; *p* value 0.001). Sharing of supervisory knowledge behavior explained 65% of the variance in organizational absorptive behavior and 87% of the variance in nurses' creativity, whereas absorptive capacity explained 55% of the variance in nurses' creativity. The lowest reliability estimates in the examined model were for the domains of digging deeper into ideas and listening to the inner voice: 0.54 (factor loading = 0.77, *p* value. 001) and 0.43 (factor loading = 0.75, *p* value 0.001), respectively. Sample size = 700. Chi-square = 1145.530. Degrees of freedom = 15. Probability level <0.001.

## 4. Discussion

Healthcare organizations should use knowledge sources and manage it in the manner that use lessons learned from the past experiences to build a robust resilient future and maintain competitiveness in turbulent environment. This future flourishes when innovation and creativity of healthcare providers especially nurses are emphasized. So, our study investigates the impact of supervisor knowledge sharing behavior and organizational absorptive capacity on nurses' creativity. It is hypothesized that supervisor knowledge sharing behavior and absorptive capacity have a significant role in stimulating nurses' creativity (the dependent variable). Our study revealed that supervisor knowledge sharing behavior and absorptive capacity are powerful determinants for nurses' creativity.

### 4.1. Supervisor Knowledge Sharing Behavior and Nurses' Creativity

Our study revealed a significant positive relationship between supervisor knowledge sharing behavior and nurses' creativity. This means that H1 of our study is accepted which give impression that sharing sources of knowledge and experiences by leaders could definitely stimulating nurses' creativity. This result is evident in our study since the majority of nurses had moderate perceived level regarding their creativity and, at the same time, had a moderate perceived level regarding the knowledge sharing behavior of their supervisors. This finding is also supported by results of regression analysis since supervisor knowledge sharing behavior accounts for 87% of variance in nurses' creativity which reflect that stimulating nurses' creativity requires a leader behavior that welcome sharing of previous experiences and dissemination of successful knowledge sources. Moreover, this reflects the high predicting power of supervisor knowledge sharing on shaping nursing creativity which means that H3 of our study is accepted. On other words, our study proved that both creative thinking and behavior among nurses could be flourished and augmented if their leaders share different sources of knowledge along with experiences in the workplace and make this sharing as pattern of improvement and communication.

This finding could be explained in the light of organizational learning theory since this theory believed that sharing of knowledge at different levels build a learning culture characterized by respecting different experiences and welcome ideas and suggestions [15]. The end result of this culture is innovation and creative ideation which is the case in our study. Also, one possible explanation of this relationship is that the knowledge sharing behavior of leaders could empower nurses in their workplace making them engaged which definitely stimulate their talents and ultimately foster the innovative and creative thinking which is the core philosophy of organization knowledge management model developed by Vespo [29]. Our finding concerning the correlation among supervisor knowledge sharing behavior and creativity is in line with the studies of Dong et al. [38], Hosseini et al. [39], Widyani et al. [40], and Revilla and Rodriguez-Prado [41]. These studies have agreed on the role of knowledge sharing among healthcare team in shaping the cognitive abilities of its members. Also, these studies found that the creative ability of staff is affected by the degree to which knowledge management process especially knowledge sharing is exercised in healthcare facility. In this context, the studies of Uyan and Şanal [12] and Kirpik and Çetin [42] found that knowledge sharing behavior in healthcare organizations could boost ambidexterity which is a metaphor for creativity and innovation which could further support our finding.

Our study proved that knowledge sharing is the most affecting factor in nurses' creativity. Moreover, knowledge sharing is a prerequisite for better performance and competitiveness gained from the creative and innovative abilities of nurses. This empirical evidence supports the ideas of Rafique and Mahmood [43] and Shaari et al. [44], who stated that knowledge sharing fosters a cohesive environment in which employees share their field-related experience, novel ideas, and job-related knowledge with one another. They learned a lot from one another and became more creative as a result. Knowledge sharing assists healthcare professionals in developing various creative approaches to patient care, thereby improving their innovative capabilities and, as a result, their work performance. Furthermore, the study of El-Sayed et al. [45] clarified that knowledge sharing behavior adopted by collaborative leaders significantly foster nurses' innovative work behavior and fuel their creativity. Conversely, the studies of Thuan [21] and Ye et al. [14] found weak role of knowledge sharing toward employees' creativity. This contradictory from our study may be due to these studies conducted on employees working in different contexts that may do repetitive tasks with high routine where knowledge growth and development are not core necessity.

It is great to find that knowledge sharing behavior is adopted by supervisors and leaders in a wide scale in our study since more than two-thirds of nurses reported that their supervisors share different sources of knowledge with them. This finding is in line with the results of Widyani et al. [40], Magnier-Watanabe and Benton [46], and Maravilhas and Martins [47] who found knowledge sharing behavior is a hallmark that characterizes the attitudes of self-managed teams. Awad et al. [48] stressed that the majority of nurses were satisfied with the valuable support received from first line nurse managers. Also, the main source of support reported by nurses is knowledge and expertise sharing. In contrary, Sodeify et al. [49] and Senek et al. [50] clarified that nurses perceived low level of the supervisors' support and low tendency to share their expertise. This may be due to poor workplace conditions that also perceived by nurses in these studies which affect their perceptions toward all positive workplace variables including support and knowledge sharing.

### 4.2. Organizational Absorptive Capacity and Nurses' Creativity

Our findings revealed significant positive relationship between absorptive capacity of organization and nurses' creativity. This means that H2 of our study is accepted which give impression that the high capacity of healthcare organization to absorb current and emerging internal and external knowledge sources is associated with high levels of nurses' creativity. This is supported by nurses' perceived levels of absorptive capacity and creativity since the majority of nurses have moderate perceived levels regarding the capacity of their organizations to absorb knowledge sources, and at the same time, they have moderate perceived levels regarding their creativity. Meanwhile, our study found that organizational absorptive capacity is a powerful antecedent for nurses' creativity since regression analysis revealed that absorptive capacity accounted for 55% of variance in nurses' creativity which means that our H4 is supported.

One possible explanation of this result is that absorptive capacity allows organizations to actively seek external sources of knowledge, such as collaboration with other firms, partnerships with research institutions, or participation in conferences. By accessing diverse knowledge, organizations are exposed to new perspectives, different ways of thinking, and alternative approaches. This exposure to diverse knowledge sparks new ideas, encourages innovation, and fuels creativity within the organization. In addition, absorptive capacity enables organizations to integrate external knowledge with their existing knowledge base. By bringing together different ideas, concepts, and perspectives, organizations can create novel combinations that often lead to innovative solutions and stimulate creativity. Our finding is the case in similar studies like Hameed et al. [51], Liu et al. [9], Asurakkody and Kim [52], and Motaghi et al. [53]. These studies found that absorptive capacity is a critical determinant for nurses' creative and innovative behavior. Conversely, the studies of Maleski et al. [54] and Fulgence et al. [27] yielded that employees' performance including the creative abilities is not significantly affected by knowledge absorptive capacity. This contradiction with our finding may be the presence of confounding factors like employees' traits, work environment, and motivation in the linkage between creativity and absorptive capacity. These confounding factors may deter absorptive capacity from exerting its role toward creativity.

### 4.3. Supervisor Knowledge Sharing Behavior and Organizational Absorptive Capacity

Our study revealed significant positive correlation between supervisor knowledge sharing behavior and absorptive capacity. This correlation is supported in our study since the majority of nurses have moderate perceived level regarding the knowledge sharing behavior of their supervisors and at the same time they have moderate perceived level regarding the capacity of their organizations to absorb, assimilate, and utilize knowledge sources for fueling innovation and creativity. Moreover, our results depict that supervisor knowledge sharing behavior accounted for 65% of variance in the absorptive capacity of organizations. This gives impression that supervisor knowledge sharing behavior is critical pivotal factor in upgrading the absorptive capacity of organizations which is necessary to sustain innovation and creativity. Meanwhile, this implied that healthcare organization could build its innovation and creativity capacity if it cultivates a culture for knowledge sharing among top leaders and employees.

Our results in this point reflect the national efforts implemented by the Egyptian state to strengthen the Egyptian human being and this could explain the results yielded in our study. Nurses in the study settings are able to access to the different sources of knowledge installed in the Egyptian knowledge bank (EKB). Meanwhile, the General Authority for Health Accreditation and Regulation (GAHAR) makes it obligatory for health facilities to share and publish health-related data in order to be eligible to universal health insurance schemes. Moreover, Internet access by healthcare providers becomes applicable and easy in the study settings after the inception of information literacy initiative taken by the Egyptian knowledge bank in collaboration with Ministry of Telecommunication. This finding is in line with the studies of Innis and Berta [55], Zhang et al. [17], and Motaghi et al. [53]. These studies reported a considerable credit for the absorptive capacity of the organizations that adopt knowledge sharing as way for improvement and sustainability.

### 4.4. Interplay between Supervisor Knowledge Sharing Behavior, Organizational Absorptive Capacity, and Nurses' Creativity

Our study revealed that absorptive capacity of organization could mediate the linkage between supervisor knowledge sharing behavior and nurses' creativity. This means nurses' creativity could ultimately be fostered if the organization builds high absorptive capacity in an environment where knowledge sharing is an absolute theme. This conclusion is consistent with Hameed et al's [51], who reported that absorptive capacity in the employability of knowledgeable workers works as a mediator between knowledge acquisition and innovation. In this context, Sancho-Zamora et al. [56], Cuevas-Vargas et al. [57], and Motaghi et al. [53] found the absorptive capacity of an organization is the key toward innovation and creativity in the presence of magnet features like knowledge sharing. All in all our study proved that in order to add value in any healthcare organization, creativity of nurses must be highlighted. Stimulating creativity requires open access for different sources of knowledge as well as enhancing the capacity to absorb different expertise and new knowledge elicited in a continuous basis.

## 5. Conclusion

Our study investigated the impact of supervisor knowledge sharing behavior and absorptive capacity toward nurses' creativity. It is concluded that supervisor knowledge sharing behavior and absorptive capacity play an important role in stimulating nurses' creativity, as supervisor knowledge sharing behavior accounted for 87% of variance in nurses' creativity and absorptive capacity accounted for 55%. Moreover, supervisor knowledge sharing behavior and absorptive capacity are powerful antecedents for nurses' creativity.

### 5.1. Implications of the Study

Based on the empirical evidence, our study provides some practical implications for nurses' managers, hospital administrations, and nurse educators. First, our study implied that modern management approaches must develop a systemic policy for knowledge management that provides a platform that maximizes individuals' capacity to learn and grow. Second, hospital directors, managers, and nursing leaders should play an important role in encouraging knowledge sharing by establishing a reward and incentive system for healthcare workers who share their knowledge with their colleagues for mutual benefit and organizational development. Third, hospital executives must communicate the benefits of knowledge sharing to employees. They must encourage and provide the necessary support, assistance, and even resources for nurses to share knowledge because knowledge and ideas sharing improves creative behavior and capabilities in organizations. Fourth, nursing school administrators should focus on developing nursing students' critical thinking skills through challenging curricula that stimulate students' high cognitive domains of thinking, resulting in future creative nurses. Fifth, stimulants and barriers of creativity in nursing schools must be taken as priority in the recent context in order to graduate nurses able to cope with technology revolution repercussions. Sixth, nursing leaders must develop creativity pathway in which talents and successful proven experiences are considered and motivated. Seventh, recruitment policies must include measures to attract leaders with a high expertise and high tendency for knowledge sharing. Finally, the selection and placement process of leaders and nurses must include tests for examining the tendency toward knowledge sharing.

### 5.2. Research Strengths and Limitations

Our study offers new insights on stimulants of nurses' creativity. It is one of the first studies that shed the light on the interplay between knowledge sharing behavior, organizational absorptive capacity, and creativity in the nursing context. Our study adds to nursing literature through providing empirical evidence about the role of knowledge sharing behavior and organizational absorptive capacity on stimulating creativity among nurses. In addition, it gives strategies and measures for nurse managers to keep their organizations competitive and sustainable in the era of globalization and intense lobar competition through acting on the creativity of nurses. Our study is one of the initiatives geared toward achieving sustainable developmental goals worldwide. Our study has high reliability as it is a multicenter study that uses robust statistical methods to ensure the validity and reliability of the instruments used. However, there are some limitations that must be recognized. Because the current findings are based on self-reported data, they are susceptible to response bias and subjectivity.

## Figures and Tables

**Table 1:** Participants' distribution according to their personal and work-related characteristics.

Demographic and professional data	No.	%
*Age (years)*		
<25	156	22.3
25–40	329	47.0
≥40	215	30.7
Mean ± SD	34.19 ± 10.16	

*Gender*		
Male	269	38.4
Female	431	61.6

*Years of experience*		
Mean ± SD	11.76 ± 10.21	
<10	347	49.6
10–20	165	23.6
≥20	188	26.9

*Qualification*		
Diploma	214	30.6
Bachelor degree	339	48.4
Specialized diploma	70	10.0
Master	62	8.9
PHD	15	2.1

*Current working unit*		
ER	145	20.7
ICU	255	36.4
NICU	136	19.4
OR	104	14.9
Inpatient units	60	8.6

*Hospital name*		
Alexandria Main University Hospital	320	45.7
Gamal Abdel Nasser Hospital	128	18.3
El Gomhoreya Public Hospital	75	10.7
Shark El-Madina Hospital	70	10.0
Mabaret Alsafra Hospital	107	15.3

**Table 2:** Participants' levels of perception regarding the study variables (supervisor knowledge sharing behavior, organizational absorptive behavior, and their creativity).

Study variables	Mean score	Low (<50%)		Moderate (50%−<75%)		High (≥75%)	
	Mean ± SD	No.	%	No.	%	No.	%
Supervisor knowledge sharing behavior questionnaire	3.66 ± 0.43	40	5.7	472	67.4	188	26.9
Supervisor explicit knowledge sharing behavior	3.57 ± 0.48	65	9.3	471	67.3	164	23.4
Supervisor tacit knowledge sharing behavior	3.91 ± 0.71	46	6.6	272	38.9	382	54.6
Organizational absorptive capacity	3.62 ± 0.41	41	5.9	523	74.7	136	19.4
Creativity	3.64 ± 0.35	18	2.6	566	80.9	116	16.6
Generating ideas	3.69 ± 0.48	42	6.0	435	62.1	223	31.9
Digging deeper into ideas	3.69 ± 0.47	39	5.6	451	64.4	210	30.0
Exploring ideas	3.52 ± 0.43	71	10.1	503	71.9	126	18.0
Listening to the inner voice	3.68 ± 0.40	20	2.9	492	70.3	188	26.9

**Table 3:** Correlation between supervisor knowledge sharing behavior, organizational absorptive behavior, and creativity.

		Supervisor knowledge sharing behavior questionnaire			Organizational absorptive capacity	Creativity				
		Supervisor explicit knowledge sharing behavior	Supervisor tacit knowledge sharing behavior	Overall		Generating ideas	Digging deeper into ideas	Exploring ideas	Listening to the inner voice	Overall
Supervisor knowledge sharing behavior questionnaire										
Supervisor explicit knowledge sharing behavior	r		0.180^*∗*^	**0.895** ^*∗*^	0.010	0.071	0.079^*∗*^	0.563^*∗*^	0.710^*∗*^	**0.420** ^*∗*^
	p		<0.001^*∗*^	**<0.001** ^*∗*^	0.788	0.060	0.038^*∗*^	<0.001^*∗*^	<0.001^*∗*^	**<0.001** ^*∗*^
Supervisor tacit knowledge sharing behavior	r			**0.599** ^*∗*^	**0.373** ^*∗*^	0.528^*∗*^	0.695^*∗*^	0.351^*∗*^	0.347^*∗*^	**0.611** ^*∗*^
	p			**<0.001** ^*∗*^	**<0.001** ^*∗*^	<0.001^*∗*^	<0.001^*∗*^	<0.001^*∗*^	<0.001^*∗*^	**<0.001** ^*∗*^
Overall	r				**0.177** ^*∗*^	0.297^*∗*^	0.379^*∗*^	0.618^*∗*^	0.735^*∗*^	**0.619** ^*∗*^
	p				**<0.001** ^*∗*^	<0.001^*∗*^	<0.001^*∗*^	<0.001^*∗*^	<0.001^*∗*^	**<0.001** ^*∗*^
Organizational absorptive capacity	r					0.765^*∗*^	0.578^*∗*^	0.252^*∗*^	0.074^*∗*^	**0.545** ^*∗*^
	p					<0.001^*∗*^	<0.001^*∗*^	<0.001^*∗*^	0.049^*∗*^	**<0.001** ^*∗*^
Creativity										
Generating ideas	r						0.764^*∗*^	0.421^*∗*^	0.206^*∗*^	**0.773** ^*∗*^
	p						<0.001^*∗*^	<0.001^*∗*^	<0.001^*∗*^	**<0.001** ^*∗*^
Digging deeper into ideas	r							0.549^*∗*^	0.415^*∗*^	**0.870** ^*∗*^
	p							<0.001^*∗*^	<0.001^*∗*^	**<0.001** ^*∗*^
Exploring ideas	r								0.747^*∗*^	**0.834** ^*∗*^
	p								<0.001^*∗*^	**<0.001** ^*∗*^
Listening to the inner voice	r									**0.714** ^*∗*^
	p									**<0.001** ^*∗*^
Overall	r									
	p									

r: Pearson coefficient. ^*∗*^Statistically significant at *p* ≤ 0.05. The bold values represent the focus of data presentation to the highly significant results.

## Data Availability

The data and materials of the current study are not publicly available due to confidentiality reason but are available from the corresponding author on reasonable request.

## Supplementary Materials

Supplementary Materials
*Supplementary File 1*. English Version of the Questionnaire: this file includes the final validated study tools used in data collection and analysis. *Supplementary File 2*. Tool Factor Analysis and Validity: this file includes the detailed testing of the study tools validity and reliability including the item factor analysis.
