# Supplementary material for: Leveraging Supervisor Knowledge Sharing Behavior and Organizational Absorptive Capacity on Nurses' Creativity
Source: J Nurs Manag. 2024 Mar 12;2024:5480761. doi: 10.1155/2024/5480761 (PMC11919019; doi:10.1155/2024/5480761)
Supplement: Supplementary Materials — Supplementary File 1. English Version of the Questionnaire: this file includes the final validated study tools used in data collection and analysis. Supplementary File 2. Tool Factor Analysis and Validity: this file includes the detailed testing of the study tools validity and reliability including the item factor analysis. [file 5480761.f1.zip › Supplementry File 1 English Version of the Questionnaire.pdf]

The Role of Supervisor Knowledge Sharing Behavior and Absorptive Capacity on Nurses Creativity

Part (1): Demographic and Professional Data

- Age:
- Gender : male ( ) female ( )
- Years of experience :
- Qualification: Diploma ( ) Bachelor degree ( ) Specialized diploma ( ) Master ( ) PHD ( )
- Current working unit: ER ( ) ICU ( ) NICU ( ) OR ( ) Inpatient wards ( )
- Hospital Name: Gamal Abdel Nasser hospital ( )
  - Alexandria main university hospital ( )
  - El Gomhoreya Public Hospital ( )
  - Shark El-Madina Hospital ( )
  - Mabaret Alsafra hospital ( )

Part (II): Supervisor knowledge sharing behavior questionnaire

Items	Strongly agree	Agree	Neutral	Disagree	Strongly disagree
1. My supervisor frequently share his/her work reports and official documents with Subordinate members in my team					
2. My supervisor always provides his/her manuals, methodologies and models for members in my team					
3. My supervisor lets me know why changes are made in work assignments					
4. My supervisor keeps me informed about official rules and policies					
5. My supervisor shares clear work instructions with me					
6. My supervisor informs me about my future work plan					
7. My supervisor informs me about my work schedule					
8. My supervisor keeps me informed about important official matters					
9. My supervisor frequently share his/her experience or know-how from work with members in my team					
10. My supervisor always provides his/her know-where or know-whom at the request of members in my team					
11. My supervisor tries to share his/her expertise from his/her education or training with members in my team					

Part (III): Absorptive capacity questionnaire

Items	Strongly agree	Agree	Neutral	Disagree	Strongly disagree
1. New opportunities to serve our clients are understood rapidly by my organization.					
2. My organization analyses and interprets changing market demands promptly.					
3. Employees in my organization record and store newly acquired knowledge for future reference.					
4. My organization quickly recognizes the usefulness of new external knowledge to existing knowledge.					
5. My organization incorporates external technological knowledge into our firm.					
6. My organization thoroughly grasps the opportunities new external knowledge offers our company					
7. In my organization employees meet periodically to discuss consequences of market trends and new product development.					
8. Employees in my organization are clearly aware of how the firm's activities should be performed					
9. My organization constantly reviews how to better exploit external knowledge.					
10. In my organization employees share a common language to refer to our products and services					

Part (VI): creativity questionnaire

Items	Strongly agree	Agree	Neutral	Disagree	Strongly disagree
1. I often find I get totally immersed in a creative idea.					
2. I am resourceful and can find the materials I need.					
3. I enjoy problem solving.					
4. I often have a strong vision for my projects.					
5. I like finding connections between things.					
6. My ideas can be odd or original.					
7. I prefer to play with ideas rather than leap on the first one.					
8. I am curious about the unknown.					
9. I find it easy to develop a strategy for a project.					
10. I find the energy and enthusiasm to research my ideas.					
11. I work persistently to complete a project.					
12. I am interested in the aim or purpose of what I am doing.					
13. The meaning of a piece of work often evolves as I work on it					
14. I don’t reject ideas with initial faults but find ways to make them work.					
15. I enjoy discovering new things.					
16. I have a sense of humor about my work					
17. I can adapt my previous skills to suit an unfamiliar task.					
18. I can reflect back on my own work.					
19. I am happy to take a risk on an idea.					
20. I enjoy working as part of a creative team.					
21. I don’t mind if ideas have more than one interpretation.					
22. I am open to my feelings about ideas.					
23. I need alone time when developing ideas.					
24. I am uninhibited when working creatively.					
25. I like ideas which people aren’t expecting.					
26. I am prepared to ignore other people’s opinions if I think my work is good.					
27. My subconscious can sometimes solve a problem when I leave it alone for a while.					
28. I have good taste and judgment about my own work and I am led by that.					
