# Supplementary material for: Leveraging Supervisor Knowledge Sharing Behavior and Organizational Absorptive Capacity on Nurses' Creativity
Source: J Nurs Manag. 2024 Mar 12;2024:5480761. doi: 10.1155/2024/5480761 (PMC11919019; doi:10.1155/2024/5480761)
Supplement: Supplementary Materials — Supplementary File 1. English Version of the Questionnaire: this file includes the final validated study tools used in data collection and analysis. Supplementary File 2. Tool Factor Analysis and Validity: this file includes the detailed testing of the study tools validity and reliability including the item factor analysis. [file 5480761.f1.zip › Supplementry File 2 Tool Factor Analysis and validity (1).pdf]

Table (1): Exploratory factor analysis (EFA) and factor loadings of Supervisor knowledge sharing behavior questionnaire

Principal Component Analysis.			
Item number	Section	Factor 1	Factor 2
item1	Part 1	0.752	
item2	Part 1	0.543	
item3	Part 1	0.752	
item4	Part 1	0.651	
item5	Part 1	0.659	
item6	Part 1	0.487	
item7	Part 1	0.682	
item8	Part 1	0.688	
item9	Part 2		0.867
item10	Part 2		0.873
item11	Part 2		0.909

The boldface indicates salient (> 0.30) loading

Table (2): Exploratory factor analysis (EFA) and factor loadings of Supervisor knowledge sharing behavior questionnaire

After rotation Promax with Kaiser Normalization				
Item number	Section	Factor 1	Factor 2	Communalities
item1	Part 1	0.761		0.590
item2	Part 1	0.531		0.417
item3	Part 1	0.761		0.590
item4	Part 1	0.647		0.526
item5	Part 1	0.653		0.539
item6	Part 1	0.475		0.464
item7	Part 1	0.691		0.589
item8	Part 1	0.696		0.592
item9	Part 2		0.870	0.757
item10	Part 2		0.876	0.768
item11	Part 2		0.913	0.833
**Kaiser-Meyer-Olkin Measure of Sampling Adequacy = 0.703**				

The boldface indicates salient (> 0.30) loading

Table (3): Exploratory factor analysis (EFA) and factor loadings of Absorptive capacity

Principal Component Analysis.			
**Item number**	**Section**	**Factor 1**	**Factor 2**
item1	Part 1	0.669	
item2	Part 1	0.641	0.700
item3	Part 1	0.714	
item4	Part 1	0.704	-0.491
item5	Part 1	0.662	
item6	Part 1	0.704	-0.491
item7	Part 1	0.654	0.690
item8	Part 1	0.617	
item9	Part 1	0.747	
item10	Part 1	0.724	

The boldface indicates salient (> 0.30) loading

Table (4): Exploratory factor analysis (EFA) and factor loadings of Absorptive capacity

After rotation Promax with Kaiser Normalization				
**Item number**	**Section**	**Factor 1**	**Factor 2**	**Communalities**
item1	Part 1	0.551	0.624	0.590
item2	Part 1	0.345	0.948	0.902
item3	Part 1	0.687	0.474	0.514
item4	Part 1	0.834		0.736
item5	Part 1	0.654	0.407	0.549
item6	Part 1	0.834		0.736
item7	Part 1	0.360	0.950	0.904
item8	Part 1	0.602	0.393	0.586
item9	Part 1	0.731	0.472	0.567
item10	Part 1	0.734	0.408	0.551
**Kaiser-Meyer-Olkin Measure of Sampling Adequacy = 0.842**				

The boldface indicates salient (> 0.30) loading

Table (5): Exploratory factor analysis (EFA) and factor loadings of Creativity

Item number	Section	Factor 1	Factor 2	Factor 3	Factor 4
		Principal Component Analysis.			
item1	Part 1	0.750		0.417	
item2	Part 1	0.725		0.391	
item3	Part 1	0.676	0.389		
item4	Part 1	0.638			
item5	Part 1	0.680	0.385		
item6	Part 1	0.601			
item7	Part 1	0.522			
item8	Part 2	0.460			
item9	Part 2	0.682	0.388	0.466	
item10	Part 2	0.651	0.516	0.335	
item11	Part 2	0.692			
item12	Part 2	0.600			
item13	Part 2	0.682	0.388	-0.466	
item14	Part 2	0.651	0.516	0.335	
item15	Part 3	0.631	0.433		
item16	Part 3		0.456		
item17	Part 3	0.683		0.451	
item18	Part 3	0.678		0.388	
item19	Part 3	0.311		0.480	
item20	Part 3	0.666			
item21	Part 3	0.522	0.363		0.494
item22	Part 4		0.556		
item23	Part 4				0.638
item24	Part 4		0.611	0.474	0.338
item25	Part 4		0.685	0.498	
item26	Part 4			0.524	0.514
item27	Part 4		0.513		0.501
item28	Part 4				0.756

The boldface indicates salient (> 0.30) loading

Table (6): Exploratory factor analysis (EFA) and factor loadings of Creativity

Item number	Section	After rotation Promax with Kaiser Normalization				
		Factor 1	Factor 2	Factor 3	Factor 4	Communalities
item1	Part 1	0.465	0.361	0.845		0.767
item2	Part 1	0.509	0.811			0.745
item3	Part 1	0.829				0.691
item4	Part 1	0.559	0.397	0.388	0.343	0.437
item5	Part 1	0.837	0.308			0.706
item6	Part 1	0.496	0.339	0.443		0.480
item7	Part 1	0.559				0.434
item8	Part 2		0.448	0.353		0.461
item9	Part 2		0.416	0.894		0.834
item10	Part 2		0.924	0.382		0.876
item11	Part 2	0.593	0.467	0.448		0.502
item12	Part 2	0.364	0.505	0.472		0.499
item13	Part 2		0.416	0.894		0.834
item14	Part 2		0.924	0.382		0.876
item15	Part 3	0.805				0.651
item16	Part 3	0.305				0.426
item17	Part 3	0.546		0.738	0.327	0.685
item18	Part 3	0.516	0.736			0.659
item19	Part 3	0.524				0.454
item20	Part 3	0.686		0.483		0.555
item21	Part 3	0.743				0.648
item22	Part 4				0.586	0.407
item23	Part 4				0.405	0.466
item24	Part 4			0.605	0.468	0.737
item25	Part 4		0.635		0.486	0.728
item26	Part 4			0.389	0.658	0.620
item27	Part 4				0.666	0.526
item28	Part 4				0.740	0.657
**Kaiser-Meyer-Olkin Measure of Sampling Adequacy = 0.853**						

The boldface indicates salient (> 0.30) loading

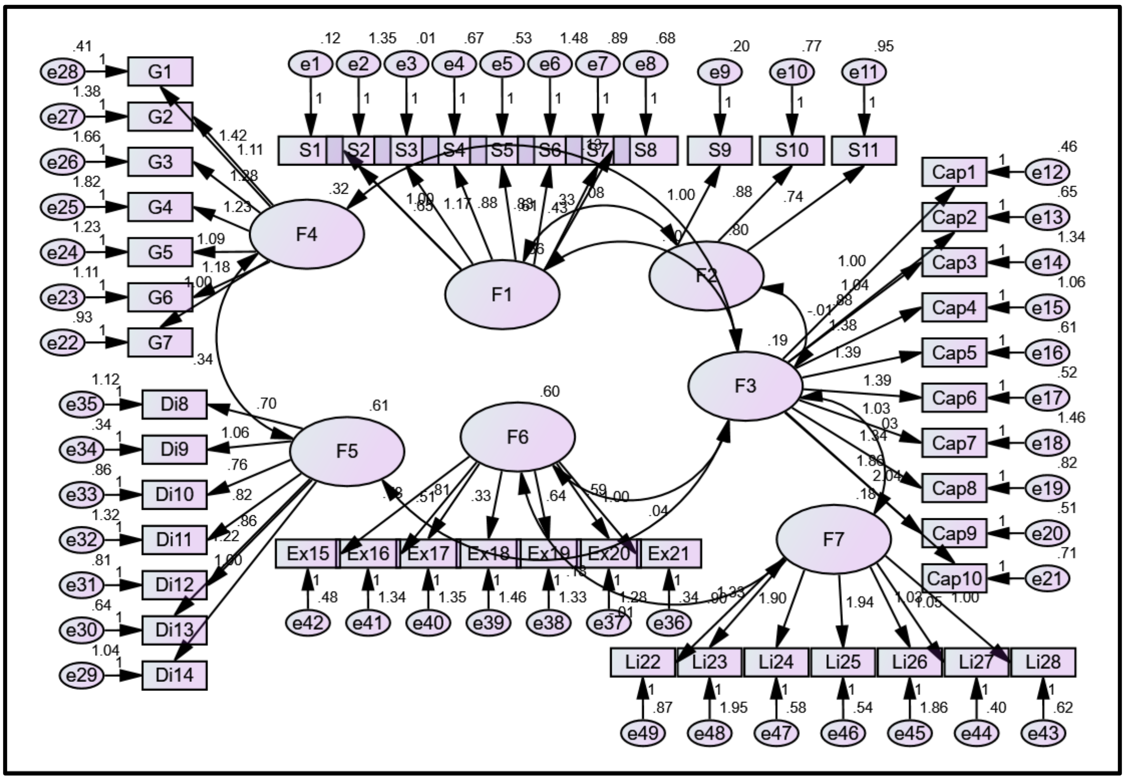

Figure (1): Confirmatory factor analysis (CFA) by Structure Equation Modeling

Tool (1): Supervisor knowledge sharing behavior questionnaire

F1 = Supervisor explicit knowledge sharing behavior
F2 = Supervisor tacit knowledge sharing behavior

F3 = **Tool (2): Absorptive capacity**

Tool (3): Creativity

F4 = Generating Ideas
F5 = Digging Deeper Into Ideas
F6 = Exploring Ideas
F7 = Listening to the Inner Voice

Model fit parameters CFI; IFI; RMSEA (1.000; 1.000; 0.117).

CFI = Comparative fit index; IFI = incremental fit index; and RMSEA = Root Mean Square Error of Approximation.
Model χ^2; significance 5.120*(0.001*)

Table (7): Corrected Item-Total Correlations for Supervisor knowledge sharing behavior questionnaire

Item number	Section	Correlation
item1	Part 1	0.665*
item2	Part 1	0.611*
item3	Part 1	0.665*
item4	Part 1	0.656*
item5	Part 1	0.662*
item6	Part 1	0.576*
item7	Part 1	0.684*
item8	Part 1	0.687*
item9	Part 2	0.895*
item10	Part 2	0.887*
item11	Part 2	0.905*

Correlation: Pearson coefficient *: Statistically significant at $p \leq 0.05$

Table (8): Corrected Item-Total Correlations for Absorptive capacity

Item number	Section	Correlation
item1	Part 1	0.657*
item2	Part 1	0.639*
item3	Part 1	0.715*
item4	Part 1	0.684*
item5	Part 1	0.661*
item6	Part 1	0.684*
item7	Part 1	0.649*
item8	Part 1	0.628*
item9	Part 1	0.763*
item10	Part 1	0.748*

Correlation: Pearson coefficient *: Statistically significant at $p \leq 0.05$

Table (9): Corrected Item-Total Correlations for Creativity

Item number	Section	Correlation
item1	Part 1	0.680*
item2	Part 1	0.725*
item3	Part 1	0.780*
item4	Part 1	0.724*
item5	Part 1	0.792*
item6	Part 1	0.664*
item7	Part 1	0.656*
item8	Part 2	0.618*
item9	Part 2	0.808*
item10	Part 2	0.827*
item11	Part 2	0.723*
item12	Part 2	0.725*
item13	Part 2	0.808*
item14	Part 2	0.827*
item15	Part 3	0.701*
item16	Part 3	0.538*
item17	Part 3	0.667*
item18	Part 3	0.668*
item19	Part 3	0.619*
item20	Part 3	0.750*
item21	Part 3	0.713*
item22	Part 4	0.655*
item23	Part 4	0.536*
item24	Part 4	0.550*
item25	Part 4	0.640*
item26	Part 4	0.608*
item27	Part 4	0.593*
item28	Part 4	0.588*

Correlation: Pearson coefficient *: Statistically significant at $p \leq 0.05$

Table (10): Cronbach's Alpha

	N of Items	Cronbach's alpha
**Tool (1):Supervisor knowledge sharing behavior questionnaire**	**11**	**0.861**
Supervisor explicit knowledge sharing behavior	8	0.766
Supervisor tacit knowledge sharing behavior	3	0.809
**Tool (2): Absorptive capacity**	**10**	**0.900**
**Tool (3): Creativity**	**28**	**0.939**
Generating Ideas	7	0.779
Digging Deeper Into Ideas	7	0.736
Exploring Ideas	7	0.792
Listening to the Inner Voice	7	0.710
